# Supplementary material for: Empirical methods for controlling false positives and estimating confidence in ChIP-Seq peaks
Source: BMC Bioinformatics. 2008 Dec 5;9:523. doi: 10.1186/1471-2105-9-523 (PMC2628906; doi:10.1186/1471-2105-9-523)
Supplement: Additional File 1 — A variety of html documents from the USeq web site detailing the available applications, their best usage, output file type descriptions, command line menus, etc. [file 1471-2105-9-523-S1.zip › USeqUserGuides/JavaDoc.zip!/JavaDoc/meme/MemeParser.html]

MemeParser


|  |  |  |
| --- | --- | --- |
| **Overview** \| **Package** \| **Class** \| **Use** \| **Tree** \| **Deprecated** \| **Index** \| **Help** | |  |
| **PREV CLASS**   **NEXT CLASS** | **FRAMES**    **NO FRAMES**     **All Classes** |  |
| SUMMARY: NESTED \| FIELD \| CONSTR \| METHOD | DETAIL: FIELD \| CONSTR \| METHOD |  |


---


## meme Class MemeParser

```
java.lang.Object
  meme.MemeParser
```

---

``` public class MemeParser extends java.lang.Object ```

Parses meme output into MemeMotif objects and stores some generalize info re the meme run.
Must supply the full command line to fire meme, use full paths for meme and the seq file.

---

**Constructor Summary**

`MemeParser(java.lang.String cndLine)`
  
          For single processor meme

`MemeParser(java.lang.String fullPathMeme,
java.lang.String fullPathFile,
java.lang.String memeParams)`
  
          For parallel processor meme on Sapo


| **Method Summary** | |
| --- | --- |
| `java.lang.String[]` | `fireMeme(java.lang.String aCmd)` |
| `java.lang.String[]` | `fireMemeParallel(java.lang.String fullPathMeme, java.lang.String fullPathFile, java.lang.String memeParams, java.lang.String fullPathToTempDir)`             Fires meme using a shell script and qsub, checks every 15 sec to see if done, returns unparsed MEME output. |
| `java.lang.String` | `getCmdLn()` |
| `MemeMotif[]` | `getMemeMotifs()` |
| `int` | `getNumMotifs()` |
| `java.lang.String` | `getParsedFileName()` |
| `void` | `parseMemeText(java.lang.String[] data)` |
| `static java.lang.String` | `reverseCompDNA(java.lang.String seq)`             takes DNA seq and reverse comps it ambiguous symbols OK. |
| `java.lang.String` | `toString()` |

**Methods inherited from class java.lang.Object**

`equals, getClass, hashCode, notify, notifyAll, wait, wait, wait`

**Constructor Detail**

### MemeParser

```
public MemeParser(java.lang.String cndLine)
```

:   For single processor meme

---


### MemeParser

```
public MemeParser(java.lang.String fullPathMeme,
                  java.lang.String fullPathFile,
                  java.lang.String memeParams)
```

:   For parallel processor meme on Sapo


**Method Detail**

### reverseCompDNA

```
public static java.lang.String reverseCompDNA(java.lang.String seq)
```

:   takes DNA seq and reverse comps it ambiguous symbols OK.
    Will warn if it finds an unrecognized base. Works with ' GATCRYWSKMBDHVNX .- '
    upper or lower case

---


### toString

```
public java.lang.String toString()
```

:   **Overrides:**: `toString` in class `java.lang.Object`

---


### parseMemeText

```
public void parseMemeText(java.lang.String[] data)
```

---


### fireMeme

```
public java.lang.String[] fireMeme(java.lang.String aCmd)
```

---


### fireMemeParallel

```
public java.lang.String[] fireMemeParallel(java.lang.String fullPathMeme,
                                           java.lang.String fullPathFile,
                                           java.lang.String memeParams,
                                           java.lang.String fullPathToTempDir)
```

:   Fires meme using a shell script and qsub, checks every 15 sec to see if done, returns unparsed MEME output.
    To be run on Sapo cluster.

    :   **Parameters:**: `fullPathMeme` - full path to the MEME program /home/sapo/software/seqanal/motifs/meme/meme.3.0.4/bin/meme: `fullPathFile` - full path to the multi FASTA for MEME to run on /home/sapo/nix/parMeme/kr4truk: `memeParams` - flags and options for the meme program -nmotifs 10 -evt 0.1 -minw 4 -maxw 12 -mod zoops -dna -revcomp -text -nostatus: `fullPathToTempDir` - full path to a directory where temporary files will be written and then deleted.

---


### getCmdLn

```
public java.lang.String getCmdLn()
```

---


### getNumMotifs

```
public int getNumMotifs()
```

---


### getMemeMotifs

```
public MemeMotif[] getMemeMotifs()
```

---


### getParsedFileName

```
public java.lang.String getParsedFileName()
```


---


|  |  |  |
| --- | --- | --- |
| **Overview** \| **Package** \| **Class** \| **Use** \| **Tree** \| **Deprecated** \| **Index** \| **Help** | |  |
| **PREV CLASS**   **NEXT CLASS** | **FRAMES**    **NO FRAMES**     **All Classes** |  |
| SUMMARY: NESTED \| FIELD \| CONSTR \| METHOD | DETAIL: FIELD \| CONSTR \| METHOD |  |


---
